# Low anterior resection syndrome after sphincter-preserving surgery for rectal cancer: predictive factors and impact on perceived functional satisfaction

**DOI:** 10.1007/s12672-026-05000-5

**Published:** 2026-04-26

**Authors:** B. Suraga, Sandeep P. Nayak, Bharath Gangadhara, Sreekanth Reddy, Ameenuddin Khan, Devaprasad Munisiddaiah, P. Vinodhini, Vidyashri H Biral, Sriram S. Janakiraman, Keerthi Ranganathan

**Affiliations:** 1 Department of Surgical Oncology, KIMS-MACS Oncosciences, KIMS Hospitals (Krishna Institute of Medical Sciences), Survey No. 36/5, Outer Ring Road, Doddanekkundi, Hobli, K.R. Puram, Mahadevapura, Bengaluru, Karnataka 560037 India; 2 Department of Surgical Oncology, Adyar Cancer Institute, 38, Sardar Patel Rd, Nehru Nagar, Adyar,, Chennai, Tamil Nadu 600036 India; 3 Department of Surgical Oncology, Fortis Hospitals, 154, 9, Bannerghatta Main Rd, Opposite IIM, Sahyadri Layout, Panduranga Nagar, Bangalore, Karnataka 560076 India

**Keywords:** Rectal cancer, Sphincter-preserving surgery, Intersphincteric resection (ISR), Low Anterior Resection Syndrome (LARS), Functional outcomes

## Abstract

**Background:**

Sphincter-preserving surgery (SPS) for rectal cancer poses challenges in balancing oncological safety with postoperative function. This study aimed to evaluate the prevalence and determinants of Low Anterior Resection Syndrome (LARS) and to examine its association with the Perceived Functional Satisfaction (Binary Assessment) following SPS.

**Methods:**

A retrospective cohort analysis was conducted among 42 patients who underwent intersphincteric resection (ISR), ultra-low anterior resection (ULAR), or low anterior resection (LAR) for rectal adenocarcinoma between July 2019 and October 2024, and completed the LARS questionnaire. Associations between LARS severity and demographic, clinical, surgical, and pathological variables were assessed.

**Results:**

Major LARS was observed in 52.38% of patients, occurring more frequently after ISR and ULAR (64.2%). Despite higher LARS severity, Perceived Functional Satisfaction remained preserved in 73.81% of patients. Major LARS was significantly associated with tumour proximity to the anal verge (*p* = 0.0265) and the SPS type (*p* = 0.0488).

**Conclusion:**

SPS achieved favourable oncological outcomes with preserved functional satisfaction. Despite a higher prevalence of LARS, Perceived Functional Satisfaction remained preserved in a substantial proportion of patients. This discordance between symptom burden and perceived satisfaction highlights the potential influence of population-specific dietary and bowel habits and underscores the need for culturally adapted functional assessment tools in rectal cancer survivorship.

## Introduction

Over the past few decades, significant advances in surgical techniques and neoadjuvant chemoradiotherapy (NACTRT) have markedly improved local control in rectal cancer, particularly for mid- and low-rectal tumors [1]. These developments have enabled a shift from traditional abdominoperineal resection (APER) to sphincter-preserving surgeries (SPS), including low anterior resection (LAR), ultralow anterior resection (ULAR), and intersphincteric resection (ISR) [2, 3]. The adoption of total mesorectal excision (TME), improved stapling devices, and enhanced recognition of key prognostic factors, such as the circumferential resection margin (CRM), have further driven this transition [2, 4, 5].

Patients who undergo LAR for rectal cancer often experience major defecation problems due to the compromised reservoir and neurosensory capacity of the rectum. Symptoms such as incontinence, increased stool frequency, and urgency are standard and collectively termed anterior or low anterior resection syndrome (LARS). The reported prevalence of LARS varies widely, ranging from 25 to 80%, depending on the definition of the syndrome, follow-up duration, and symptom severity. Several studies suggest that a low anastomotic level is a key risk factor for LARS [1, 6].

The first formal definition of LARS was proposed in 2012, characterizing it as bowel dysfunction following rectal resection that reduces quality of life (QoL). In 2020, an international consensus further refined this definition, incorporating patient-reported outcomes. While earlier tools, such as the Cleveland Clinic Florida Fecal Incontinence Score (CCFIS) or the Wexner score, focused primarily on incontinence, the LARS score has since become the standard due to its comprehensive assessment of symptom severity and its impact on QoL. It classifies patients into three categories: no LARS, minor LARS, or major LARS. Today, up to 50% of long-term rectal cancer survivors report LARS symptoms, significantly affecting their QoL [5, 7].

Notably, most existing LARS-related literature originates from Western populations, with limited data from the Indian subcontinent. Differences in dietary patterns, bowel habits, sociocultural expectations, and adaptive behaviors may influence symptom perception and tolerance, potentially affecting patient-reported functional outcomes. The applicability and interpretability of standardized LARS scoring systems in such settings, therefore, remain uncertain, highlighting a critical gap in region-specific survivorship research.

Against this background, the present study aims to assess the prevalence and severity of LARS among Indian patients undergoing sphincter-preserving surgery for rectal cancer and to evaluate its association with clinicopathological variables, surgical techniques, oncological outcomes, and patient-reported functional satisfaction. By contextualizing LARS within an Indian population, this study seeks to contribute regionally relevant data and inform culturally sensitive approaches to postoperative functional assessment and survivorship care.

## Methods

### Study design and patient selection

#### Study design

This retrospective cohort study was conducted at a tertiary oncology center and included patients who underwent curative-intent surgery for histologically confirmed rectal adenocarcinoma between July 2019 and October 2024. Ethical clearance was obtained from the Institutional Ethics Committee at Fortis Hospital. The study adhered to the STROBE guidelines and the Declaration of Helsinki (2013) and used anonymized data with no additional interventions. All patients were counseled appropriately and provided written informed consent. Data confidentiality and privacy were maintained throughout the study.

#### Inclusion criteria

Patients aged 18 years or older with histologically confirmed rectal adenocarcinoma located below the peritoneal reflection were eligible for inclusion. Only those who underwent curative-intent SPS, including ISR, LAR, or ULAR, and who completed the LARS questionnaire were included to ensure the availability of functional outcome data. Adequate follow-up data for both oncological and functional assessments were required for inclusion.

**Fig. 1:**
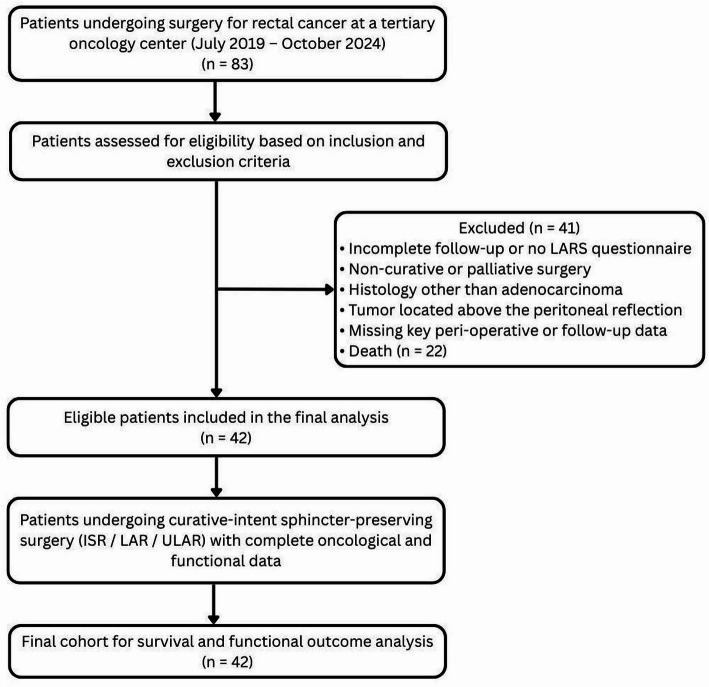
STROBE-compliant flow diagram of patient selection

During the study period, a total of 83 patients underwent surgery for rectal cancer. Among the cohort, 42 patients completed follow-up, provided responses to the LARS evaluation, and were thus included in the final analysis. Peri-operative data were available for all 83 patients; however, survival and functional analyses were restricted to those with complete data (Fig. 1).

#### Exclusion criteria

Patients were excluded if they had incomplete follow-up, died during the follow-up period (*n* = 22), or did not respond to the LARS questionnaire, underwent non-curative or palliative procedures, had histology other than adenocarcinoma, had tumors located above the peritoneal reflection, or had missing key peri-operative or follow-up data that precluded meaningful analysis (Fig. 1).

STROBE-compliant flow diagram depicting patient selection for this retrospective cohort study. Of 83 patients assessed, 42 met eligibility criteria and were included in the final analysis.

### Surgical approach

Procedures were performed using minimally invasive surgery. The surgical procedure was selected based on tumour location and patient-specific features. Although robotic-assisted surgery was generally preferred for ISR and ULAR, and the laparoscopic approach was preferred for LAR, the final choice of technique was individualised based on patient-specific factors and preferences. All procedures were conducted by experienced colorectal surgeons, adhering to oncological principles including TME.

### Perioperative management

Neoadjuvant treatment was administered according to multidisciplinary tumour board recommendations and included long-course NACTRT, short-course radiotherapy (SCRT), or total neoadjuvant therapy (TNT) in selected cases. A minority of patients underwent upfront surgery. Temporary diverting ileostomies were created in most patients and reversed once anastomotic healing was confirmed, at the treating team’s discretion. As per our institutional protocol, a diverting stoma was routinely created for all patients who received neoadjuvant radiotherapy and for those undergoing low rectal resections, even when surgery was performed upfront. This practice aims to minimise the risk of anastomotic complications in high-risk anastomoses.

### Functional outcome assessment

Postoperative bowel function was assessed using the validated LARS score. The LARS questionnaire was administered in person or by phone at least 6 months after stoma reversal, allowing bowel function to stabilise. Patients were classified as having no LARS (0–20), mild LARS (21–29), or major LARS (30–42) using conventional criteria.

Previous studies in Western populations have consistently demonstrated that patients with major LARS experience significantly poorer QoL. In the present study, a formal QoL assessment using validated instruments was not feasible. Consequently, a simplified Perceived Functional Satisfaction (Binary Assessment) was developed to capture patients’ overall self-reported perception of postoperative well-being and its impact on daily life. This measure was intended as a pragmatic, exploratory assessment rather than a substitute for validated QoL assessments [8, 9, 10].

### Data collection

Clinical data were obtained from institutional electronic medical records and operative databases. Extracted variables included demographic details, neoadjuvant treatment, surgical technique, pathological findings, time to stoma closure, anastomotic complications, and long-term oncological outcomes.

## Statistical analysis

Associations between categorical variables were assessed using the Fisher–Freeman–Halton exact test, as appropriate. A p-value < 0.05 was considered statistically significant. Non-significant associations are presented in tabular form.

To explore factors associated with LARS occurrence (presence vs. absence), additional regression analyses were conducted. Univariable logistic regression was conducted for clinically relevant variables, followed by multivariable logistic regression to assess independent associations. Effect estimates are reported as odds ratios (OR) with 95% confidence intervals (CI).

For categorical analyses, selected variables were recategorized a priori to facilitate statistical testing. Stoma reversal timing was treated as a categorical variable, classified as early reversal (≤ 30 days), standard reversal (30–60 days), or late reversal (> 60 days) [11, 12]. Pathological T stage was dichotomized into early (Tis–T2) and advanced (T3–T4) stages, while nodal status was grouped as node-negative (N0) and node-positive (N1–N2). Anastomotic complications were defined as a composite variable comprising anastomotic leak and conduit necrosis. Patients receiving either NACTRT or SCRT were collectively categorized as having received neoadjuvant therapy.

All analyses were performed using Epi Info version 7.2.6.0 and Python and R on Google Colab.

## Results

### Demographic and clinical profile

The study comprised 42 rectal cancer patients with an average age of 58.76 ± 14.01 years, with a predominance of males (73.81%). Tumours were primarily located in the lower rectum (52.38%), and the most common surgical approach was robotic (64.29%). ISR was performed in 45.24% of patients, followed by LAR (33.33%) and ULAR (21.43%) (Table 1).

**Table 1:** Demographic and clinical profile

S.No	Variables		Value
1	Age (Mean ± SD)		58.76 ± 14.01
2	Gender (n, %)	Male	31 (73.81%)
		Female	11 (26.19%)
3	Tumour Site (n, %)	Lower Rectum	22 (52.38%)
		Mid Rectum	20 (47.62%)
4	Surgical Approach (n, %)	Robotic	27 (64.29%)
		Laparoscopic	14 (33.33%)
		Conversion to Open	1 (2.38%)
5	Type of Surgery (n, %)	ISR	19 (45.24%)
		LAR	14 (33.33%)
		ULAR	9 (21.43%)

^*ISR* Intersphincteric Resection, *LAR* Low Anterior Resection, *ULAR* Ultra−Low Anterior Resection^

### Operative and postoperative details

The median hospital stay was 5.00 (4.00–7.00) days. Mean intraoperative blood loss was 345.71mL. Stomas were created in 92.86% of cases, with a median reversal time of 45.00 (28.00-174.00) days. LARS was recorded after a median interval of 590.50 (339.00−943.00) days post-stoma reversal. Tumours were within 5 cm of the anal verge (distance from AV) in 59.52% of patients (Table 2).

**Table 2:** Operative and Postoperative Details

S.No	Variables	Value
1	Hospital Stay in days (Median, IQR)	5.00 (4.00–7.00)
2	Blood Loss in mL (Mean ± SD)	345.71 ± 218.23
3	Stoma Created (n, %)	39 (92.86%)
4	Days to Stoma Reversal (Median, IQR)	45.00 (28.00–174.00)
5	LARS recording interval post stoma reversal in days (Median, IQR)	590.50 (339.00–943.00)
6	Tumour within 5 cm of anal verge (n, %)	25 (59.52%)

^*IQR* Interquartile Range, *SD* Standard Deviation, *LARS* Low Anterior Resection Syndrome^

### Pathological characteristics

Pathological complete response (pCR) was observed in 14.29% of patients who received neoadjuvant therapy. Most tumours were Grade 2 (61.90%). The TME quality was deemed complete in nearly all cases (97.62%), with only one patient (2.38%) showing CRM involvement. The T3 stage was the most common (38.10%), with 54.76% of cases being node-negative. The mean harvested lymph nodes was 10.50 ± 4.30, with a median of 1.00 (1.00–2.00) positive nodes. Anastomotic leaks and conduit necrosis were observed in 3 patients each (7.14%), with no overlap between these complications (Table 3).

**Table 3:** Pathological characteristics

S.No	Variables		Value
1	Histology (n, %)	Adenocarcinoma	39 (92.86%)
		Mucinous Adenocarcinoma	1 (2.38%)
		Intramucosal adenocarcinoma (TVA with HGD)	1 (2.38%)
		Signet Ring Cell Carcinoma	1 (2.38%)
2	Tumour Grade (n, %)	pCR	6 (14.29%)
		1	7 (16.67%)
		2	26 (61.90%)
		3	3 (7.14%)
3	TME Grading (n, %)	Complete	41 (97.62%)
		Incomplete	1 (2.38%)
4	T stage (n, %)	pCR	6 (14.29%)
		Tis/T0	3 (7.14%)
		T1	3 (7.14%)
		T2	13 (30.95%)
		T3	16 (38.10%)
		T4	1 (2.38%)
5	N stage (n, %)	pCR	6 (14.29%)
		N0	23 (54.76%)
		N1	11 (26.19%)
		N2	2 (4.76%)
6	Involved Resection Margin Status (n, %)	Free	41 (97.62%)
		Circumferential	1 (2.38%)
7	Tumour Invasion (n, %)	LVI	2 (4.76%)
		PNI	3 (7.14%)
8	Lymph Nodes	Harvested (Mean ± SD)	10.50 ± 4.30
		Positive (Median, IQR)	1.00 (1.00–2.00)
9	Anastomotic Complications (n, %)	Anastomotic leak	3 (7.14%)
		Conduit Necrosis	3 (7.14%)

*TVA* Tubulovillous adenoma, *HGD* High grade dysplasia, *pCR* Pathological Complete Response, *TME* Total Mesorectal Excision, *LVI* Lymphovascular Invasion, *PNI* Perineural Invasion, *SD* Standard Deviation, *IQR* Interquartile Range

### Clinical and oncological outcomes

The clinical and oncological outcomes of the study cohort are summarised in Table 4. Regarding neoadjuvant treatment, the majority of patients received NACTRT (25 patients, 59.52%), followed by SCRT (12 patients, 28.57%), while 5 patients (11.90%) underwent upfront surgery without neoadjuvant treatment. Pathological response assessment demonstrated a pCR in 6 patients (14.29%), near-complete response in 4 patients (9.52%), partial response in 21 patients (50.00%), and poor response in 6 patients (14.29%); treatment effect assessment was not applicable in 5 patients who underwent upfront surgery (11.90%).

Functional outcomes analysis revealed a median LARS score of 30.50 (IQR: 26.00–36.00). Based on LARS severity grading, 9 patients (21.43%) reported no LARS, 11 patients (26.19%) experienced minor LARS, and 22 patients (52.38%) had major LARS. Despite the high prevalence of major LARS, 31 patients (73.81%) reported perceived functional satisfaction with preserved function, assessed using a binary measure (Table 4).

**Table 4:** Clinical outcomes

S.No	Variables		Value
A. Oncological Outcomes			
1	Type of Neoadjuvant Treatment (n, %)	NACTRT	25 (59.52%)
		SCRT	12 (28.57%)
		None	5 (11.90%)
2	Treatment Effect (n, %)	pCR	6 (14.29%)
		Near Complete Response	4 (9.52%)
		Partial Response	21 (50.00%)
		Poor Response	6 (14.29%)
		Not Applicable	5 (11.90%)
B. Functional Outcomes			
1	LARS	(Median, IQR)	30.50 (26.00–36.00)
		No (n, %)	9 (21.43%)
		Minor (n, %)	11 (26.19%)
		Major (n, %)	22 (52.38%)
2	Perceived Functional Satisfaction with Preserved Function (n, %)		31 (73.81%)

*NACTRT* Neoadjuvant Chemoradiotherapy, *SCRT* Short−course Radiotherapy, *pCR* Pathological Complete Response, *IQR* Interquartile Range, , *LARS* Low Anterior Resection Syndrome

### Statistical associations

A total of 50 statistical comparisons were performed across demographic, clinicopathological, surgical, and treatment-related variables. Of these, 6 associations were statistically significant (*p* < 0.05), whereas the remaining comparisons did not reach statistical significance.

#### Factors affecting LARS

Significant associations between surgical and tumor-related variables and LARS outcomes are presented in Table 5. Major LARS was significantly associated with SPS type, with higher rates observed following ISR and ULAR, and LAR (*p* = 0.0488). In addition, tumors located within 5 cm of the anal verge were significantly associated with the development of Major LARS (*p* = 0.0265).

Anastomotic complications were not significantly associated with the development of Major LARS. Major LARS occurred in 19 of 36 patients (52.77%) without anastomotic complications and in 3 of 6 patients (50.0%) with complications (*p* > 0.05) (Table 5).

The incidence of Major LARS was higher among patients who received neoadjuvant radiotherapy compared with those who underwent upfront surgery without radiotherapy. Major LARS was observed in 8 of 12 patients (66.67%) treated with SCRT, in 13 of 25 patients (52.0%) treated with NACTRT, and in 1 of 5 patients (20.0%) who underwent upfront surgery. This difference did not reach statistical significance (*p* = 0.1745). Comparisons between radiotherapy subgroups (NACTRT vs. SCRT, NACTRT vs. upfront surgery, and SCRT vs. upfront surgery) also did not demonstrate statistically significant differences (all *p* > 0.05). No significant associations were observed between Major LARS and nodal stage, demographic variables, or neoadjuvant therapy (Table 5).

**Table 5:** Statistical associations between LARS severity and clinical parameters and outcomes

Clinical association (*n* = 42)		*n* (%)	Major LARS		Minor LARS		Overall LARS (Major + Minor)	
			*n* (%)	*p*	*n* (%)	*p*	*n* (%)	*p*
Age	Middle-aged (< 60 years)	20 (47.62%)	7 (35%)	0.0623^a^	8 (40%)	0.0806^a^	15 (75%)	0.7139^a^
	Elderly (> 60 years)	22 (52.38%)	15 (68.18%)		3 (13.64%)		18 (81.82%)	
Gender	Male	31 (73.81%)	17 (54.84)	0.7298^a^	7 (22.58%)	0.4369^a^	24 (77.42%)	1^a^
	Female	11 (26.19%)	5 (45.45%)		4 (36.36%)		9 (81.82%)	
Type of surgery	ISR and ULAR	28 (66.67%)	18 (64.29%)	**0.0488** ^**a**^ *	7 (25%)	1^a^	25 (89.29%)	**0.0407** ^**a**^ *
	LAR	14 (33.33%)	4 (28.57%)	**0.0488** ^**a**^ *	4 (28.57%)	1^a^	8 (57.14%)	**0.0407** ^**a**^ *
Timing of Stoma Reversal	Early Stoma Reversal	10 (23.81%)	7 (70%)	0.2842^a^	1 (10%)	0.2451^a^	8 (80%)	1^a^
	Standard Stoma Reversal	11 (26.19%)	5 (45.45%)	0.7298^a^	3 (27.27%)	1^a^	8 (72.73%)	0.6756^a^
	Late Stoma Reversal	18 (42.86%)	10 (55.56%)	0.7638^a^	6 (33.33%)	0.4832^a^	16 (88.89%)	0.2578^a^
Distance from AV		25 (59.52%)	17 (68%)	**0.0265** ^**a**^ *	6 (24%)	0.7327^a^	23 (92%)	**0.0191** ^**a**^ *
Anastomotic Complications		6 (14.29%)	3 (50%)	1^a^	2 (33.33%)	0.6437^a^	5 (83.33%)	1^a^
Neoadjuvant Therapy	Overall	37 (88.10%)	21 (56.76%)	0.1745^a^	9 (24.32%)	0.5931^a^	30 (81.08%)	0.2881^a^
	NACTRT Therapy	25 (59.52%)	13 (52%)	1^a^	6 (24%)	0.7327^a^	19 (76%)	0.7164^a^
	SCRT	12 (28.57%)	8 (66.67%)	0.3148^a^	3 (25%)	1^a^	11 (91.67%)	0.2475^a^
	Upfront Therapy	5 (11.90%)	1 (20%)	0.1745^a^	2 (40%)	0.5931^a^	3 (60%)	0.2881^a^
Perceived Functional Satisfaction		31 (73.81%)	16 (51.61%)	0.1658^a^	10 (32.26%)	0.2342^a^	26 (83.87%)	1^a^

*LARS* Low Anterior Resection Syndrome, *ISR* Intersphincteric Resection, *ULAR* Ultra−Low Anterior Resection, *LAR* Low Anterior Resection, *Distance from AV* Tumor within 5 cm of anal verge, *NACTRT* Neoadjuvant Chemoradiotherapy, *SCRT* Short−course Radiotherapy, a Fisher’s Test, * Significant values

These trends suggest potential relationships that may become statistically significant in larger cohorts and warrant further evaluation in adequately powered studies. Importantly, no trends were observed in the Tumor or Nodal stage or demographic variables, suggesting limited influence of these factors on LARS outcomes in the present cohort.

#### Independent clinical associations with major LARS

To evaluate whether the factors identified in Table 5 represented independent associations, variables that were statistically significant in Fisher’s exact test were further analyzed using binary logistic regression. Neoadjuvant therapy was also included because of its established biological relevance in post-treatment bowel dysfunction.

In univariable logistic regression, ISR and ULAR procedures were associated with a higher odds ratio (OR = 6.25) for developing LARS than LAR (*p* = 0.03). Similarly, tumors located within 5 cm of the anal verge were associated with increased odds (OR = 8.05) of LARS (*p* = 0.02). Neoadjuvant therapy showed a trend toward increased LARS but did not reach statistical significance (*p* = 0.30) (Table 6).

No statistical significance was found in the multivariable logistic regression model. However, the direction of effect remained consistent with the univariable analysis, with ISR and ULAR (OR = 2.25) and tumors located within 5 cm of the anal verge (OR = 4.62) continuing to demonstrate higher odds ratios (Table 6).

**Table 6:** Univariate and multivariate analysis of factors associated with LARS

SL.NO	Parameter	OR	95% CI	*p* Value
Univariable analysis				
1	Distance from AV	8.05	1.42–45.77	0.02*
2	Neoadjuvant Therapy	2.86	0.40–20.47	0.30
3	Type of surgery - ISR and ULAR	6.25	1.26–30.90	0.03*
Multivariable analysis				
1	Distance from AV	4.62	0.53–40.03	0.17
2	Neoadjuvant Therapy	1.81	0.19–17.31	0.61
3	Type of surgery - ISR and ULAR	2.25	0.29–17.61	0.44

^*OR* Odds Ratio, *CI* Confidence Interval, *Distance from AV* Tumor within 5 cm of anal verge, *ISR* Intersphincteric Resection, *ULAR* Ultra−Low Anterior Resection^

^*Significant values^

#### Relationship between LARS severity and perceived functional satisfaction (binary assessment)

**Fig. 2:**
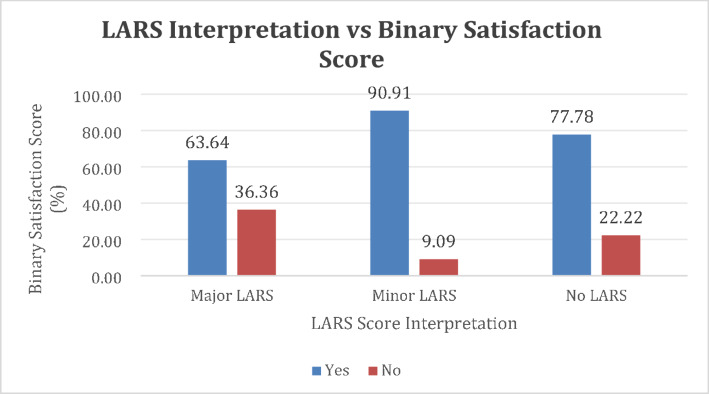
LARS score interpretation vs. perceived functional satisfaction (binary assessment)

Figure 2 presents the relationship between LARS Score Interpretation and Perceived Functional Satisfaction. Perceived Functional Satisfaction was reported in 90.91% (10/11) of patients with minor LARS, 77.78% (7/9) with no LARS, and 63.64% (14/22) with major LARS.

Bar graph showing the percentage of patients with preserved Perceived Functional Satisfaction across LARS severity levels.

Despite this trend toward a decrease in Perceived Functional Satisfaction with increasing LARS severity, no statistically significant association was found between LARS categories and Perceived Functional Satisfaction. Fisher’s exact test showed non-significant p-values for major LARS (*p* = 0.1658), minor LARS (*p* = 0.2342), and no LARS (*p* = 1.0000). These findings indicate that, although lower Perceived Functional Satisfaction was more common among patients with major LARS, the differences did not reach statistical significance (Table 5).

#### Comparison between LARS questionnaire responders and non-responders

Baseline demographic, clinical, and treatment-related characteristics were compared between responders and non-responders to the LARS questionnaire to assess cohort comparability and potential non-response bias. No statistically significant differences were observed between the two groups with respect to gender (*p* = 0.6295), distance from the anal verge (*p* = 0.361), surgical approach including ISR/ULAR (*p* = 0.1222) or LAR (*p* = 0.1222), neoadjuvant therapy (*p* = 0.3628), or nodal stage (*p* = 0.1923). Tumor T stage showed a borderline difference between responders and non-responders; however, this did not reach statistical significance (*p* = 0.0728) (Table 7).

**Table 7:** Statistical associations between LARS responders and clinical parameters and outcomes

Clinical Association (*n* = 83)		*n* (%)	LARS Response	
			*n* (%)	*p*
Age	Middle-aged (< 60 years)	34 (40.96%)	20 (58.82%)	0.2663^a^
	Elderly (> 60 years)	49 (59.04%)	22 (44.90%)	
Gender	Male	58 (69.88%)	31 (53.45%)	0.6295^a^
	Female	24 (28.92%)	11 (45.83%)	
Type of surgery	ISR and ULAR	48 (57.83%)	28 (58.33%)	0.1222^a^
	LAR	35 (42.17%)	14 (40%)	0.1222^a^
Distance from AV		41 (49.40%)	25 (60.98%)	0.361^a^
Neoadjuvant Therapy		66 (79.52%)	37 (56.06%)	0.3628^a^
T Stage	Early Stage	29 (34.94%)	19 (65.52%)	0.0791^a^
	Advanced Stage	38 (45.78%)	17 (44.74%)	
N Stage	Node Negative	40 (48.19%)	23 (57.50%)	0.2021^a^
	Node Positive	27 (32.53%)	13 (48.15%)	

*LARS* Low Anterior Resection Syndrome, *ISR* Intersphincteric Resection, *ULAR* Ultra−Low Anterior Resection, *LAR* Low Anterior Resection, *Distance from AV* Tumor within 5 cm of anal verge, a Fisher’s Test

## Discussion

### Principal findings

SPS for rectal cancer aims to achieve oncological adequacy while maintaining postoperative bowel function and QoL [13]. This study highlights the delicate balance between these objectives and identifies clinical and pathological variables closely linked to both LARS severity and oncologic outcomes. Among 42 patients undergoing ISR, ULAR, or LAR, functional preservation was achieved.

### LARS severity and patient-reported satisfaction

Major LARS occurred in 52.38% of patients, consistent with previous studies reporting a high incidence of bowel dysfunction following low anastomoses [14, 15]. Interestingly, no statistically significant association was observed between LARS and Perceived Functional Satisfaction, replicating prior studies that found no correlation between overall QoL and LARS, which is attributed to patients’ adaptability and acceptance of postoperative changes [16, 17, 18].

Although patients with major LARS typically report lower QoL in the global literature [19], our cohort demonstrated preserved Perceived Functional Satisfaction (73.81%) despite higher rates of major LARS among those undergoing ULAR and ISR (64.2%). This suggests that functional impairment does not necessarily translate into diminished subjective well-being. Notably, ISR is often evaluated using the Wexner score, which focuses solely on faecal incontinence. In contrast, our study utilised the LARS score, which encompasses a broader range of postoperative bowel function domains, providing a more comprehensive assessment of functional outcomes [20, 21].

Additionally, strong family support, social adaptation, and flexible toileting practices may enhance tolerance to bowel dysfunction and mitigate its perceived burden [17]. Many patients also prioritised oncologic cure over functional limitations, reporting satisfaction despite symptoms. Furthermore, the Perceived Functional Satisfaction assessment tool used may not have captured more nuanced decrements in well-being.

### Contextual considerations and adaptation

The preservation of Perceived Functional Satisfaction observed in this cohort, even among patients with severe LARS, may in part reflect underlying cultural and dietary influences. Most existing literature on LARS and QoL has originated from Western populations, where dietary patterns and bowel habits differ substantially [19]. The traditional Indian diet, naturally high in fibre, promotes softer stools and more regular bowel movements. Furthermore, daily defecation is culturally regarded as usual in Indian settings, whereas bowel movements as infrequent as three times per week are considered normal in Western populations [22, 23, 24]. Consequently, bowel frequency patterns classified as abnormal by Western scoring systems may be physiologically typical and less distressing to Indian patients.

These differences underscore the importance of developing culturally adapted assessment tools for evaluating postoperative function and QoL in rectal cancer survivors. Our findings emphasise the need for caution when applying the LARS scoring system in diverse populations, particularly in the Indian context, where baseline bowel habits and perceptions of normalcy differ. The substantial weighting of frequency and clustering symptoms in the current LARS score may not accurately reflect functional severity or its impact on Perceived Functional Satisfaction in this population.

There are very few studies that have examined the outcomes of SPS among Indian patients. Therefore, a culturally and population-specific validation study is warranted to determine whether recalibrating the LARS score, potentially by modifying symptom weightings, would be necessary to more accurately reflect functional outcomes and Perceived Functional Satisfaction in this setting.

### Surgical approach and technical considerations

Robotic-assisted approaches were more commonly used for ISR and ULAR, whereas LAR was predominantly performed laparoscopically. However, the surgical approach was individualised, based on the patient’s anatomy, tumour characteristics, and personal preference. The robotic platform’s enhanced dexterity and visualisation offer distinct advantages in the confined pelvic cavity, facilitating high-quality TME, particularly in patients with a narrow pelvis [25]. In this cohort, complete TME was achieved in 97.62% of resections, with only one case of CRM involvement underscoring the technical precision achievable with minimally invasive SPS. These results align with prior studies, which have shown comparable oncologic outcomes between robotic and laparoscopic approaches, with improved functional preservation and reduced morbidity [26].

### Stoma reversal and functional outcomes

Previous studies have identified stoma closure time as a potential determinant of LARS severity, as delayed reversal may impair neorectal adaptation [1]. Xia et al. [27] proposed a cut-off of 128 days, beyond which the risk of major LARS rises significantly. In the present study, no statistically significant association was observed between stoma reversal timing and LARS severity across early, standard, and late closure groups (all *p* > 0.25) (Table 5).

When absolute distributions were examined, Major LARS was observed in 7 of 10 patients (70.0%) in the early reversal group, 5 of 11 patients (45.5%) in the standard reversal group, and 10 of 18 patients (55.6%) in the delayed reversal group. Although these proportions did not demonstrate a consistent gradient favouring earlier closure, the absence of statistically significant differences suggests that, within the relatively early median closure timeframe of this cohort (45.00 (28.00–174.00) days), stoma reversal timing alone may not be a dominant determinant of functional outcome. The heterogeneity observed across groups likely reflects the influence of multiple interacting factors, including sphincter-preserving technique, radiotherapy exposure, and individual adaptive capacity (Table 5).

In interpreting functional outcomes following stoma reversal, the potential for non-response bias must be considered. In this cohort, baseline demographic, clinicopathological, and treatment-related characteristics were comparable between patients who completed the LARS questionnaire and those who did not, suggesting that the reported functional outcomes are representative of the overall SPS population and are unlikely to be substantially influenced by differential response related to stoma status or disease severity (Table 7).

### Clinical implications

The findings of this study underscore the importance of systematic postoperative functional assessment following SPS for rectal cancer. Although major LARS was common, patient-reported Perceived Functional Satisfaction (Binary Assessment) was frequently preserved, highlighting that symptom severity and global patient satisfaction may not fully overlap, and supporting the need for culturally adapted functional assessment tools in the Indian context. These results support the need for individualized patient counselling that addresses both functional expectations and adaptive capacity rather than relying solely on symptom-based scoring systems. In retrospective or resource-constrained settings, simplified patient-reported measures may provide preliminary insight into patient experience; however, they should complement, rather than replace, validated functional and QoL instruments.

### Limitations

This study has several significant limitations. Its retrospective, single-centre design and modest sample size limit statistical power and preclude causal inference. The use of a Perceived Functional Satisfaction (Binary Assessment) rather than validated QoL instruments limits comparability with the existing international literature. It may fail to capture subtle or multidimensional impairments in postoperative well-being. Selection bias related to non-response cannot be excluded, and LARS assessment at variable time points following stoma reversal may have introduced heterogeneity in functional outcomes. Additionally, the absence of preoperative functional assessment limits evaluation of postoperative change.

From a statistical standpoint, the number of outcome events was limited, particularly the small number of patients without LARS. Consequently, the univariable and multivariable logistic regression analyses were underpowered and produced wide confidence intervals, indicating imprecision and model instability. The regression results are therefore interpreted as exploratory associations rather than definitive independent predictors. These limitations should be carefully considered when interpreting the results.

## Conclusion

In conclusion, LARS remains a frequent functional consequence of SPS for rectal cancer, particularly following low anastomoses. However, the severity of LARS was not statistically significantly associated with patient-reported Perceived Functional Satisfaction (Binary Assessment) in this cohort, suggesting that postoperative adaptation and subjective well-being may not be fully captured by symptom-based scoring alone. While these findings should be interpreted cautiously, given the study’s limitations, they highlight the complexity of functional outcome assessment and the need for culturally sensitive, validated tools in future prospective studies to evaluate patient-centred outcomes after rectal cancer surgery more accurately.

## Data Availability

The data that support the findings of this study are available from the corresponding author upon reasonable request. The data are not publicly available because doing so could compromise the privacy of research participants.

## References

[1] Benli S, Çolak T, Türkmenoğlu MÖ. Factors influencing anterior/low anterior resection syndrome after rectal or sigmoid resections. Turk J Med Sci. 2021;51(2):623–30. 10.3906/sag-2007-145. 33078605 10.3906/sag-2007-145 PMC8203143

[2] Luvisetto F, Shamali A, Rutgers MLW, Flashman K, Khan JS. Sphincter preservation in patients with low rectal cancer: striking the right oncological balance. Discov Oncol. 2021;12(1):7. 10.1007/s12672-021-00400-1. 33855312 10.1007/s12672-021-00400-1 PMC7976658

[3] Zheng K, Hu Q, Yu G, Zhou L, Yao Y, Zhou Y, Wang H, Hao L, Yu E, Lou Z, Zhang Y, Qiu H, Meng R, Zhang W. Trends of sphincter-preserving surgeries for low lying rectal cancer: A 20-year experience in China. Front Oncol. 2022;12:996866. 10.3389/fonc.2022.996866. 36568186 10.3389/fonc.2022.996866 PMC9773833

[4] Takemasa I. Advances and controversies in treatment for locally advanced rectal cancer over the past decades: West meets East. Ann Gastroenterol Surg. 2020;4(4):314–5. 10.1002/ags3.12371. 32724873 10.1002/ags3.12371 PMC7382423

[5] Keane C, Fearnhead NS, Bordeianou LG, Christensen P, Basany EE, Laurberg S, Mellgren A, Messick C, Orangio GR, Verjee A, Wing K, Bissett I, LARS International Collaborative Group. International Consensus Definition of Low Anterior Resection Syndrome. Dis Colon Rectum. 2020;63(3):274–84. 32032141 10.1097/DCR.0000000000001583 PMC7034376

[6] Ryoo SB. Low anterior resection syndrome. Ann Gastroenterol Surg. 2023;7(5):719–24. 10.1002/ags3.12695. 37663958 10.1002/ags3.12695 PMC10472409

[7] Trabelsi M, Samaali I, Kammoun N, ben Safta A, Oueslati A, Dougaz W, Khalfallah M, Jerraya H, Bouasker I, Nouira R, Dziri C. Predictive factors of major low anterior resection syndrome after surgery for rectal tumors. Tunis Med. 2024;102(10):702–7. 39441154 10.62438/tunismed.v102i10.5177 PMC11574385

[8] Ketelaers SHJ, van Heinsbergen M, Orsini RG, Vogelaar FJ, Konsten JLM, Nieuwenhuijzen GAP, Rutten HJT, Burger JWA, Bloemen JG. Functional Bowel Complaints and the Impact on Quality of Life After Colorectal Cancer Surgery in the Elderly. Front Oncol. 2022;12:832377. 35242714 10.3389/fonc.2022.832377 PMC8886503

[9] Zhang R, Luo W, Qiu Y, Chen F, Luo D, Yang Y, He W, Li Q, Li X. Clinical Management of Low Anterior Resection Syndrome: Review of the Current Diagnosis and Treatment. Cancers (Basel). 2023;15(20):5011. 10.3390/cancers15205011. Erratum in: Cancers (Basel). 2024;16(2):459. 10.3390/cancers16020459. 10.3390/cancers15205011 PMC10605930 37894378

[10] Plastiras A, Korkolis D, Frountzas M, Theodoropoulos G. The effect of anastomotic leak on postoperative pelvic function and quality of life in rectal cancer patients. Discov Oncol. 2022;13(1):52. 10.1007/s12672-022-00518-w. 35751713 10.1007/s12672-022-00518-w PMC9233722

[11] Ellebæk MB, Perdawood SK, Steenstrup S, Khalaf S, Kundal J, Möller S, Bang JC, Støvring J, Qvist N. Early versus late reversal of diverting loop ileostomy in rectal cancer surgery: a multicentre randomized controlled trial. Sci Rep. 2023;13(1):5818. 10.1038/s41598-023-33006-4. 37037856 10.1038/s41598-023-33006-4 PMC10085999

[12] Podda M, Coccolini F, Gerardi C, Castellini G, Wilson MSJ, Sartelli M, Pacella D, Catena F, Peltrini R, Bracale U, Pisanu A. Early versus delayed defunctioning ileostomy closure after low anterior resection for rectal cancer: a meta-analysis and trial sequential analysis of safety and functional outcomes. Int J Colorectal Dis. 2022;37(4):737–56. 10.1007/s00384-022-04106-w. 35190885 10.1007/s00384-022-04106-w PMC8860143

[13] Pappou EP, Temple LK, Patil S, Smith JJ, Wei IH, Nash GM, Guillem JG, Widmar M, Weiser MR, Paty PB, Schrag D, Garcia-Aguilar J. Quality of life and function after rectal cancer surgery with and without sphincter preservation. Front Oncol. 2022;12:944843. 10.3389/fonc.2022.944843. 36353560 10.3389/fonc.2022.944843 PMC9639454

[14] Emmertsen KJ, Laurberg S, Rectal Cancer Function Study Group. Impact of bowel dysfunction on quality of life after sphincter-preserving resection for rectal cancer. Br J Surg. 2013;100(10):1377-87. 10.1002/bjs.9223. 10.1002/bjs.9223 23939851

[15] Croese AD, Lonie JM, Trollope AF, Vangaveti VN, Ho YH. A meta-analysis of the prevalence of Low Anterior Resection Syndrome and systematic review of risk factors. Int J Surg. 2018;56:234–41. 10.1016/j.ijsu.2018.06.031. 29936195 10.1016/j.ijsu.2018.06.031

[16] Bohlok A, Mercier C, Bouazza F, Galdon MG, Moretti L, Donckier V, El Nakadi I, Liberale G. The burden of low anterior resection syndrome on quality of life in patients with mid or low rectal cancer. Support Care Cancer. 2020;28(3):1199–206. 10.1007/s00520-019-04901-2. 31218414 10.1007/s00520-019-04901-2

[17] Laursen BS, Sørensen GK, Majgaard M, Jensen LB, Jacobsen KI, Kjær DK, Juul T, Christensen P, Mikkelsen AH. Coping strategies and considerations regarding low anterior resection syndrome and quality of life among patients with rectal cancer; a qualitative interview study. Front Oncol. 2022;12:1040462. 10.3389/fonc.2022.1040462. 36523984 10.3389/fonc.2022.1040462 PMC9745191

[18] Jacob KC, Muralee AT, Sudham M, Balakrishnan MWLM. Low Anterior Resection Syndrome and Quality of Life of Patients After Sphincter Preservation Surgery: A Prospective Study. Cureus. 2024;16(5):e60059. 10.7759/cureus.60059. 38860066 10.7759/cureus.60059 PMC11162878

[19] Pieniowski EHA, Nordenvall C, Palmer G, Johar A, Tumlin Ekelund S, Lagergren P, Abraham-Nordling M. Prevalence of low anterior resection syndrome and impact on quality of life after rectal cancer surgery: population-based study. BJS Open. 2020;4(5):935–42. 32530135 10.1002/bjs5.50312 PMC7528525

[20] Zhang B, Zhuo GZ, Zhao Y, Zhao YJ, Zhu J, Liu FF, Ding JH. Quality of Life and Functional Outcomes After Intersphincteric Resection for Ultralow Rectal Cancer: A Prospective Observational Study. Dis Colon Rectum. 2023;66(7):1029–1038. doi: 10.1097/DCR.0000000000002615. 10.1097/DCR.0000000000002615 36602458

[21] Alvandipour M, Karami MY, Azadfar M, Yazdani Charati J. Inter sphincter rectal resection with and without Malone ante grade continence enema in cases with low rectal cancer: A randomized, prospective, single-blind, clinical trial. Casp J Intern Med. 2022 Summer;13(3):546–54. 10.22088/cjim.13.3.546 PMC9348201 35974933

[22] Panigrahi MK, Kar SK, Singh SP, Ghoshal UC. Defecation frequency and stool form in a coastal eastern Indian population. J Neurogastroenterol Motil. 2013;19(3):374–80. 10.5056/jnm.2013.19.3.374. 23875105 10.5056/jnm.2013.19.3.374 PMC3714416

[23] Walker AR, Walker BF, Bhamjee D, Walker EJ, Ncongwane J, Segal I. Defaecation frequencies in Black, Indian, Coloured and White populations - what do they signify? S Afr Med J. 1982;62(7):195–9. 6285528

[24] Singh P, Surana R, Soni S, Agnihotri A, Ahuja V, Makharia GK, Staller K, Kuo B. Cross cultural comparison of constipation profiles at tertiary care centers between India and USA. Neurogastroenterol Motil. 2018 Mar 9. 10.1111/nmo.13324. 10.1111/nmo.13324 29521026

[25] Huang CW, Tsai HL, Yeh YS, Su WC, Huang MY, Huang CM, Chang YT, Wang JY. Robotic-assisted total mesorectal excision with the single-docking technique for patients with rectal cancer. BMC Surg. 2017;17(1):126. 10.1186/s12893-017-0315-x. 29208050 10.1186/s12893-017-0315-x PMC5716256

[26] Evans KM, Sahawneh JM, Ferrara M. Rectal cancer surgery: is robotic surgery supported by solid evidence? Ann Laparosc Endosc Surg. 2023;8:14. 10.21037/ales-22-76.

[27] Xia F, Zou Y, Zhang Q, Wu J, Sun Z. A novel nomogram to predict low anterior resection syndrome (LARS) after ileostomy reversal for rectal cancer patients. Eur J Surg Oncol. 2023;49(2):452–60. 10.1016/j.ejso.2022.10.015. 37406079 10.1016/j.ejso.2022.10.015

